# Spectator medicine at an international mega sports event: Rugby World Cup 2019 in Japan

**DOI:** 10.1186/s12199-020-00914-0

**Published:** 2020-11-24

**Authors:** Takuya Tajima, Yuji Takazawa, Mutsuo Yamada, Takuro Moriya, Haruhiko Sato, Junichiro Higashihara, Yukimasa Toyama, Etsuo Chosa, Akihiko Nakamura, Ichiro Kono

**Affiliations:** 1 Rugby World Cup 2019 Organising Committee, Tokyo, Japan; 2 Division of Orthopaedic Surgery, Department of Medicine of Sensory and Motor Organs, Faculty of Medicine, University of Miyazaki, 5200 Kihara, Kiyotake, Miyazaki, 889-1692 Japan; 3 Department of Sports Medicine and Sportology, Graduate School of Medicine, Health and Sports Science, Juntendo University, Tokyo, Japan; 4 Faculty of Health and Sports Sciences, Ryutsu Keizai University, Ryugasaki, Japan; 5 Department of Orthopaedic Surgery, Chiba Rosai Hospital, Ichihara, Japan; 6 Department of Neurosurgery, Seirei Mikatahara General Hospital, Hamamatsu, Japan; 7 Department of Gynecology, Higashihara Clinic, Fukuoka, Japan; 8 Toyama Orthopaedic Clinic, Osaka, Japan; 9 Nakamura Surgery and Pediatrics Clinic, Tokyo, Japan; 10 University of Tsukuba, Tsukuba, Japan

**Keywords:** Spectator medicine, Wet bulb globe temperature, Heat stroke, Rugby World Cup, Patient presentation rate

## Abstract

**Background:**

The Rugby World Cup (RWC) is one of the biggest international mega sports events in the world. This study was conducted to identify and evaluate the volume, nature, and severity of spectator medical care in the stadiums of 12 venues across Japan during RWC 2019.

**Method:**

This was a retrospective review of medical records from spectator medical rooms of 45 official matches of RWC 2019 between September 20 and November 2, 2019. All patients in the stadium who visited the spectator medical room and were transferred to a hospital were included. The wet bulb globe temperature (WBGT) value at the kick-off time of each match, the number of visits to the spectator medical room, and the number of transfers to a hospital were reviewed and analyzed. The patient presentation rate (PPR) was calculated per 10,000 attendees. Severity categories were defined as mild or severe. Mild cases were considered non-life threatening requiring minimal medical intervention, and severe cases required transport to a hospital.

**Result:**

The total number of visits to the spectator medical room was 449 with a PPR of 2.63. Most cases (91.5%) were mild in severity. The PPR was significantly higher for the matches held with a WBGT over 25 °C than for the matches under 21 °C (PPR 4.27 vs 2.04, *p* = 0.04). Thirty-eight cases were transferred to a hospital by ambulance; the PPR was 0.22. The most common reasons for transfer to the hospital were heat illness and fracture/dislocation, at a rate of 15.8% each. The incidence rate of cardiopulmonary arrest per 10,000 attendees was 0.0059 during RWC 2019.

**Conclusion:**

Preparation and provision of appropriate medical service for spectators is a key factor for mass-gathering events. During RWC 2019, the majority (91.5%) of patients who sought medical attention did so for minor complaints, which were easily assessed and managed. On the other hand, a higher WBGT situation contributes significantly to an increased PPR (< 21 versus > 25, 2.04 versus 4.27, *p* = 0.04). Careful medical preparation, management, and development of public education programs for higher WBGT situations will be required in the future for similar international mega sports events.

## Background

The Rugby World Cup (RWC) is one of the biggest international mega sports events in the world. The 9th RWC took place in Japan from September 20 to November 2, 2019. During these 44 days, a total of 45 international matches were played in 12 venues across Japan, but, unfortunately, 3 matches were canceled due to a typhoon disaster. This competition was the very first event in the history of the RWC to be held in Asia. The total number of spectators in the stadiums was 1,704,443 (with roughly 242,000 foreign spectators); the average number of spectators per match was 37,877; and the ticket sales rate was 99.3% [1].

The medical support system in the field of play and the medical service for players and match officials are strictly managed and supervised by the World Rugby, including the required international medical licenses, such as “Immediate Care in Rugby” level 2 or 3 certified by the World Rugby or “Pre-Hospital Immediate Care in Sports” level 2 or 3 certified by the Rugby Football Union (England) [2]. On the other hand, the medical service system for spectators depended on the RWC Organising Committee (OC).

The definition of a mass gathering event has traditionally been a group of over 1000 attendees gathered at a specific location for a specific period. Medical services for spectators are key issues for mass gathering events. Arbon reported that a mass gathering event is a situation during which a gathering of crowds produces limited access to patients; thus, there is a potential for delayed response to a medical emergency [3]. Moreover, there is the potential risk of alcohol-related problems at rugby events. Several papers have reported that some factors predicted patient presentation rates (PPRs) at mass gathering events, and the most common patient presentations at mass gathering events were mild in severity [4, 5]. On the other hand, Milsten et al. reported that it was important to integrate the planning within the existing emergency medical care system, given that routine emergency care must continue [6].

The Medical Advisory Group Medical Officers (MAGMO) team of the RWC 2019 OC was established in March 2017. The MAGMO team was finally composed of 8 members: one tournament medical director (TMD), three original members, and 4 additional members appointed in April 2019. One member of the MAGMO team had to take charge and be responsible for medical issues in the stadium for each of the 45 matches.

MAGMO sought to address the organization of the medical system for every medical issue of the RWC, including pitch side care management, team medical support, and camp area medical support, as well as spectator medicine. The present study was developed to provide a descriptive analysis of medical preparation and support for RWC 2019 spectators.

## Materials and methods

Preparation for the establishment of spectator medicine

1. Number of spectator medical rooms and physicians in the stadium

The maximum capacity and the number of actual seats of each of the 12 venues were reviewed. Moreover, the characteristics of the previous events in these 12 stadiums and the number of spectator medical rooms were also confirmed. Referring to the mass gathering guideline of Tokyo [7], one medical room with one physician and one nurse for up to 10,000 people, an additional physician and nurse for every additional 10,000 people, the number of spectator medical rooms, and the plan for the number of medical staff were organized (Table 1). This medical room for spectators was available from the spectator gate opening time, 3 h before kick-off, to closing time in the stadium, 90 min after the final whistle of the match.

2. Medical kit for spectator medicine

**Table 1:** Characteristics of each venue

Venue	Maximum capacity	No. of sales seats	No. of spectator medical room	Remarks (previous event and No. of medical room)
Sapporo	41,000	36,900–38,950	2	Asian games 2017, 1
Kamaishi	16,000	14,400–15,200	2	New stadium
Kumagaya	24,000	21,600–22,800	2	National athletic competition, 1
Tokyo	49,000	44,100–46,550	3	National athletic competition, 3
Yokohama	71,000	63,900–67,450	4	FIFA 2002, 4
Shizuoka	50,000	45,000–47,500	4	Professional soccer league, 5
Toyota	42,000	37,800–39,900	3	Rugby international club match, 2
Hanazono	23,000	20,700–21,850	2	National high school Rugby tournament, 1
Kobe	28,000	25,200–26,600	2	FIFA 2002, 4
Fukuoka	20,000	18,000–19,000	2	Rugby international test match, 1
Kumamoto	30,000	27,000–28,500	2	Rugby international test match, 2
Oita	39,000	35,100–37,050	3	FIFA 2002, 4

*FIFA 2002* 2002 Fédération Internationale de Football Association

Basically, the role of spectator medical staff was providing first-aid and minimal medical care. However, severe cases were sometimes recognized in the stadium and required moderate to advanced treatment and assessment, such as cardiopulmonary resuscitation (CPR) or transport to a hospital by ambulance. Referring to the medical kit of spectator medicine of previous national athletic competitions in Japan, MAGMO reviewed and selected the medical kit for spectator medicine.

3. Documentation

It was necessary to record each event and treatment in the medical room. Referring to the medical document sheet of the medical rooms of previous national athletic competitions in Japan, MAGMO reviewed and established simple clinical recording documentation for the spectator medical room (Table 2).

4. Ambulance

**Table 2:** The clinical medical form for spectator medical care

		Clinical record form: Spectator medical room		
**Stadium**		**( )**		
**Spectator med**			**Date**	
**room number**			**Time**	
**Situation**		**During the game ( )**	**Location**	
		**in motion ( )**		
		**Others ( )**		
**Patient information**		**Name**	**Sex**	**Male / Female**
		**DOB**	**Age**	**( )years old**
		**Contact No.**		
**Diagnosis / Symptom**		**Gastrointestinal disorder (include vomiting or abdominal pain)** **Fever, Cold, Anemia, Headache, Heat illness, Tooth problem Fatigue, Eye problem, Ear problem, Consciousness disorder, Respiratory discomfort, Dizziness, Chest pain, Bruise, Sprain Fracture, Dislocation, Rupture, Wound (stratch, abrasion, cut)**		
		**Location of injury ( )**		
		**Others ( )**		
**Critical cause** **Past history/ allergy**				
**Treatment**		**Time ( : )**		
**Medical kit used in Treatment**				
**Others**				
**Contact**	**Address**			
	**cell No**			
**Ambulance**		**(Yes ・ No)**		
**Date: / / Physician's Name:**				

Usually, it is very difficult for an official ambulance to remain in the stadium for hours in Japan. Therefore, the RWC 2019 OC, in association with local government and the emergency response command teams of the 12 venues, asked for and obtained one official ambulance for spectators in the stadium area for each match.

5. Translation system for foreign spectators

Rugby football is an international sport, and 20 teams from various countries and regions attended RWC 2019. Basically, RWC 2019 OC, MAGMO, and the area medical officer selected medical staff who could speak and understand English to be in charge of the spectator medical room. However, spectators were not only from English-speaking areas, but also from other language areas. Therefore, to overcome the language barriers, a remote translation system (Towarow, Towa Engineering Co., Tokyo, Japan) for foreign spectators was introduced in the medical room. This system could translate by a professional translator from Japanese to 6 other languages (English, Chinese, Spanish, French, Russian, and Italian) using a tablet or smartphone. This translation service was available from the spectator gate opening time to closing time in the stadium.

6. Measurement of wet bulb globe temperature (WBGT) at kick-off time
  Outdoor events have been expected to have a higher incidence of environmental illnesses, such as heat illness. Usually, high temperatures and humidity are seen in September in Japan [8]. Therefore, the WBGT value was measured and/or recorded referring to the weather report for the surrounding area from the website provided by the Japan Meteorological Agency at the kick-off time of each match. The measurement was carried out by MAGMO using appropriate instruments at playing field level.
7. Access to the medical room

Over 150 volunteers in each stadium, police, and security staff patrolled the stadium during the match. When they discovered an injured or ill person, that person was brought to the nearest spectator medical room.

8. Infection control

Basically, MAGMO established an infection control program according to the Ministries of Health, Labour and Welfare [9] and National Institute of Infectious Diseases [10] for both teams and travelers before the competition.

### Study design

**Fig. 1:**
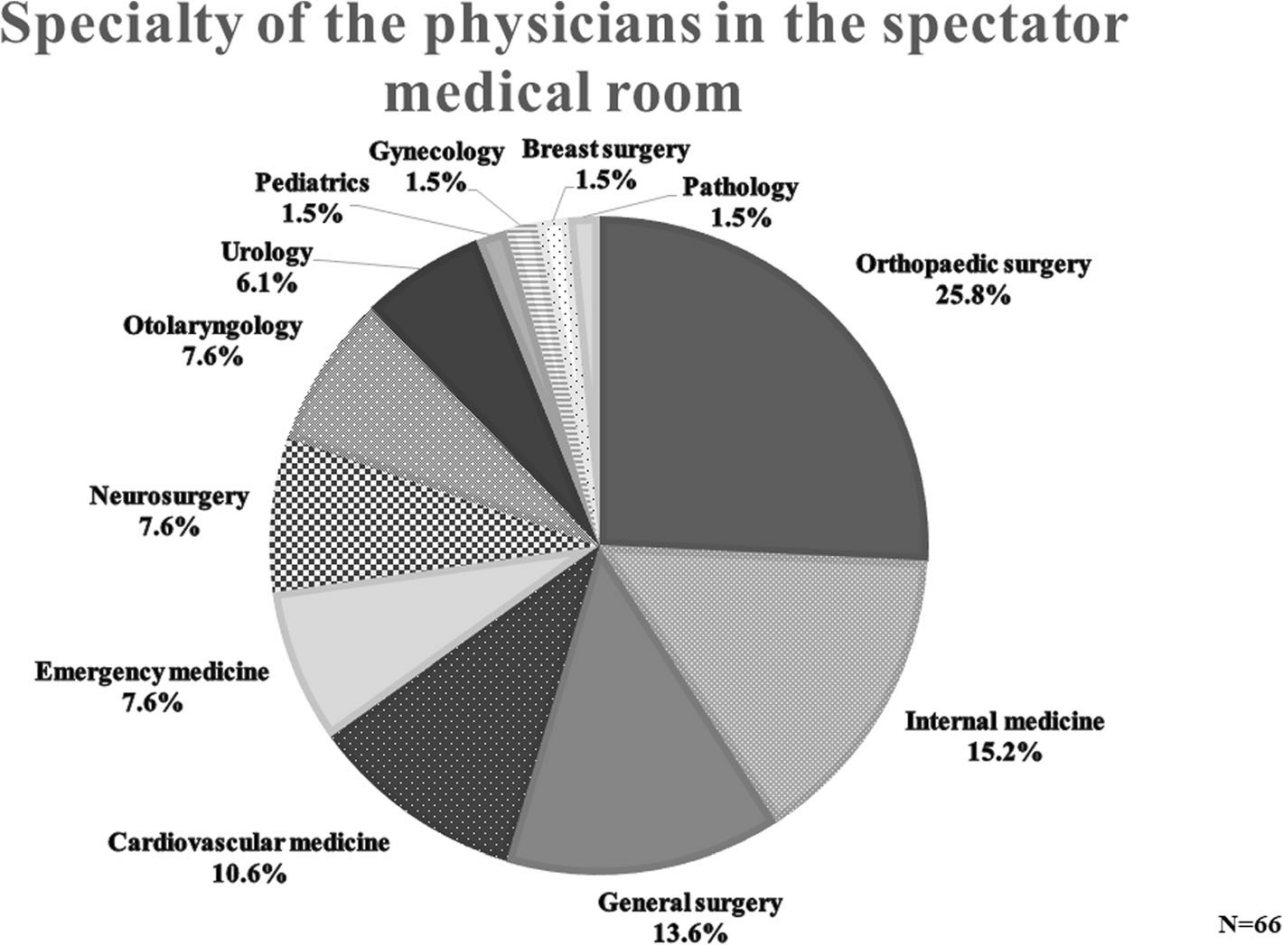
The proportions of specialties of physicians in the spectator medical room. These physicians were selected by the area medical officer based on clinical ability and experience

The study followed a retrospective, observational design including data collected during the competitive period of RWC 2019, with the opening match on September 20, 2019, and the final match on November 2, 2019, with a total of 45 matches. Finally, the number of physicians in the spectator medical rooms was 66, with a cumulative total number of physicians of 134. The specialties of these 66 physicians were as follows: orthopaedic surgery 17 (25.8%); internal medicine 10 (15.2%); general surgery 9 (13.6%); cardiovascular medicine 7 (10.6%); emergency medicine, neurosurgery, and otolaryngology 5 each (7.6%); urology 4 (6.1%); and paediatrics, gynecology, breast surgery, and pathology 1 each (1.5%) (Fig. 1). The number of nurses in the spectator medical rooms was 85, and the cumulative total number of nurses was 134. Each physician in the spectator medical room prepared and recorded the documentation for each case and the daily report. The venue MAGMO was responsible for collecting and reporting the documents to TMD after every match. Therefore, the response rate for these documents was 100% in RWC 2019. The full documents were reviewed by TMD and MAGMO. Inclusion criteria were cases with full documentation from the spectator medical rooms during the RWC 2019 in the above period.

The WBGT value at the kick-off time of each match, the number of visits to the spectator medical rooms including the number of foreign spectators, the number of transfers to the hospital by ambulance, details of the numbers and incidence rates of diagnosis/symptoms of patient presentation, and the proportions of diagnosis/symptoms of the cases that were transferred to the hospital were reviewed and analyzed. The PPR was calculated and expressed as per 10,000 attendees. Severity categories were defined as mild and severe. Mild cases were considered non-life-threatening and required minimal medical intervention, whereas severe cases required transport to a hospital. The age and sex of participants and the time of visit to the spectator medical room were also reviewed.

The Poisson distribution was used for calculation of 95% confidence intervals (CIs); a result was considered significant if the 95% CIs did not overlap. The Kruskal-Wallis test was performed for non-parametric analyses of more than 3 groups. Chi-squared testing was performed for the relationship between the age and sex of the participants and for the sex and time analysis. Statistical analyses were performed using the statistical software package BellCurve for Excel 2015 (Social Survey Research Information Co., Ltd., Tokyo, Japan). The level of significance was set at *p* < 0.05.

## Results

The WBGT value at kick-off time is shown in Table 3; seven matches were played with a WBGT temperature over 25 °C: match number (MN) 16 in Kumagaya, MN 17 in Tokyo, MN 33 in Shizuoka, MNs 11 and 19 in Fukuoka, and MNs 20 and 24 in Oita (Table 3). The total number of visits to the spectator medical rooms was 449 (male 217, female 232) in the 45 matches, with an average of 9.98 cases per match (range 2–25 cases per match), and the PPR was 2.63 per 10,000 attendees. Of these cases, 73 cases (16.3%, PPR 0.42 per 10,000 attendees) were foreign spectators who could not speak and understand Japanese. The total number of foreign spectators was roughly 242,000, and the PPR per 10,000 foreign spectators was 3.02. All 12 venues had foreign spectator visits to the medical room. Thirty-eight cases (male/female 25/13) were transferred from the stadium to the hospital by ambulance; the PPR was 0.22 per 10,000 attendees (Table 4). The PPR of the matches held with a WBGT over 25 °C (4.27 per 10,000 attendees) was significantly higher than that of matches played at a WBGT under 21 °C (2.04 per 10,000 attendees, *p* = 0.04) (Table 5). The number of spectators transferred to a hospital was 17 (PPR 0.2) with WBGT < 21 °C, 13 (PPR 0.2) with WBGT of 21 to 25 °C, and 8 (PPR 0.37) with WBGT > 25 °C, with no significant difference (Table 5).

**Table 3:** Match number and WBGT value (°C) at kick-off time

Venue											
Sapporo	Kamaishi	Kumagaya	Tokyo	Yokohama	Shizuoka	Toyota	Hanazono	Kobe	Fukuoka	Kumamoto	Oita
(2) 17.2	(10) 19.1	(9) 22.3	(1) 19.5	(4) 19.8	(14) 22.8	(8) 23.3	(5) 21.6	(12) 22.8	(11) 25.0	(28) 24.8	(20) 26.2
(7) 13.2		(16) 35.0	(3) 20.6	(6) 23.1	(23) 24.0	(15) 20.6	(13) 22.6	(18) 24.9	(19) 26.0	(39) 19.8	(24) 25.5
		(30) 19.8	(17) 28.8	(40) 17.3	(31) 22.0	(26) 19.1	(21) 24.0	(22) 22.3	(36) 20.0		(32) 24.2
			(25) 21.9	(45) 19.0	(33) 26.0		(38) 21.0	(29) 19.0			(41) 23.1
			(27) 21.1	(46) 19.0							(43) 21.2
			(42) 19.6	(48) 17.8							
			(44) 20.2								
			(47) 18.8								

(Official match number), (34), (35), and (37) were canceled due to typhoon

*WBGT* wet bulb globe temperature (°C)

**Table 4:** Number of visits to the spectator medical room

	Venue													
	Sapporo	Kamaishi	Kumagaya	Tokyo	Yokohama	Shizuoka	Toyota	Hanazono	Kobe	Fukuoka	Kumamoto	Oita	Total	PPR
No. of match	2	1	3	8	6	4	3	4	4	3	2	5	45	
Total No. of visits	11	8	44	90	72	57	24	37	25	18	10	53	449	2.63
No. of foreign countries spectator	3	1	2	23	12	6	1	2	8	3	2	10	73	0.42
No. of transfer to the hospital	1	0	2	6	6	4	3	4	5	1	0	6	38	0.22

*PPR* patient presentation rate per 10,000 attendees

**Table 5:** Patient presentation depend on WBGT category

WBGT (°C)	No. of match	Total number of spectator	Total number of visits to the spectator medical room	PPR	Total number of transfer to the hospital	Transfer to the hospital rate per 10,000 attendees
< 21	19	834,338	170	2.04*	17	0.20
21–25	19	654,687	187	2.86	13	0.20
25 <	7	215,418	92	4.27*	8	0.37

*PPR* patient presentation rate per 10,000 attendees

Kruskal-Wallis test for non-parametric analysis

*p = 0.04

**Fig. 2:**
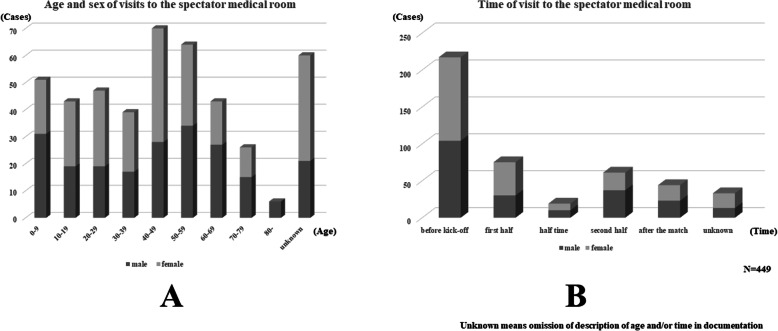
Characteristics of participants. **a** Age and sex of participants who visited the spectator medical room. The age and sex distribution were follows: 0–9 years, 51 cases (male 31, female 20); 10–19 years, 43 cases (male 19, female 24); 20–29 years, 47 cases (male 19, female 28); 30–39 years, 39 cases (male 17, female 22); 40–49 years, 70 cases (male 28, female 42); 50–59 years, 64 cases (male 34, female 30); 60–69 years, 43 cases (male 27, female 16); 70–79 years, 26 cases (male 15, female 11); ≥ 80 years, 6 cases (all males); and unknown, meaning no description of age in the documentation, 60 cases (male 21, female 39). There is no significant relationship between age and sex. **b** Time of visit to the spectator medical room. The time of the participants’ visits to the spectator medical rooms were as follows: before kick-off, 217 cases (male/female, 104/113); first half, 75 cases (30/45); half time, 19 cases (10/9); second half, 61 cases (37/24); after the match, 44 cases (23/21); and unknown category, which means no description of time in the documentation, 33 cases (13/20). There is no significant relationship between sex and time of visit

The patient presentations in the spectator medical rooms were wound/scratch/abrasion in 84 cases (18.7%); heat illness in 52 (11.6%); gastrointestinal disorder in 49 (10.9%); bruise/sprain in 49 (10.9%); headache in 35 (7.8%); dizziness in 33 (7.3%); fever in 26 (5.8%); fatigue in 15 (3.3%); and consciousness disorder (including due to alcohol) in 12 (2.7%) (Table 6). Ten fracture/dislocation cases (2.2%), 5 chest pain cases (1.1%), and 5 respiratory discomfort cases (1.1%) were also confirmed. The incidence rates per 10,000 attendees and 95% CIs are also shown in Table 6. Most cases were mild in severity (91.5%). The age and sex distribution of users of the medical rooms is shown in Fig. 2a. Forty to 49 years was the largest number with 70 cases (male 28, female 42). There was no significant relationship between the age and the sex of the participants. The time of the participants’ visits to the spectator medical rooms are shown in Fig. 2b. Largest numbers of participants were visited before kick-off, 217 cases (male/female, 104/113). There was no significant relationship between sex and time of visit.

**Table 6:** Details of patient presentation in spectator medicine

Diagnosis/symptoms	No. of patients	incidence / 10,000 attendees	95% Confidence Intervals
Wound/scratch/abrasion	84	0.49	0.39–0.66
Heat illness	52	0.31	0.15–0.71
Gastrointestinal disorder	49	0.29	0.21–0.40
Bruise/sprain	49	0.29	0.20–0.39
Headache	35	0.21	0.10–0.25
Dizziness	33	0.19	0.12–0.32
Fever	26	0.15	0.08–0.27
Fatigue	15	0.09	0.01–0.18
Consciousness disorder (include due to alcohol)	12	0.07	0.02–0.15
Fracture/dislocation	10	0.06	0.01–0.11
Chest pain	5	0.03	0.00–0.06
Respiratory discomfort	5	0.03	0.00–0.07
Others	74	0.43	0.24–0.74
Total	449	2.63	2.35–3.50

**Fig. 3:**
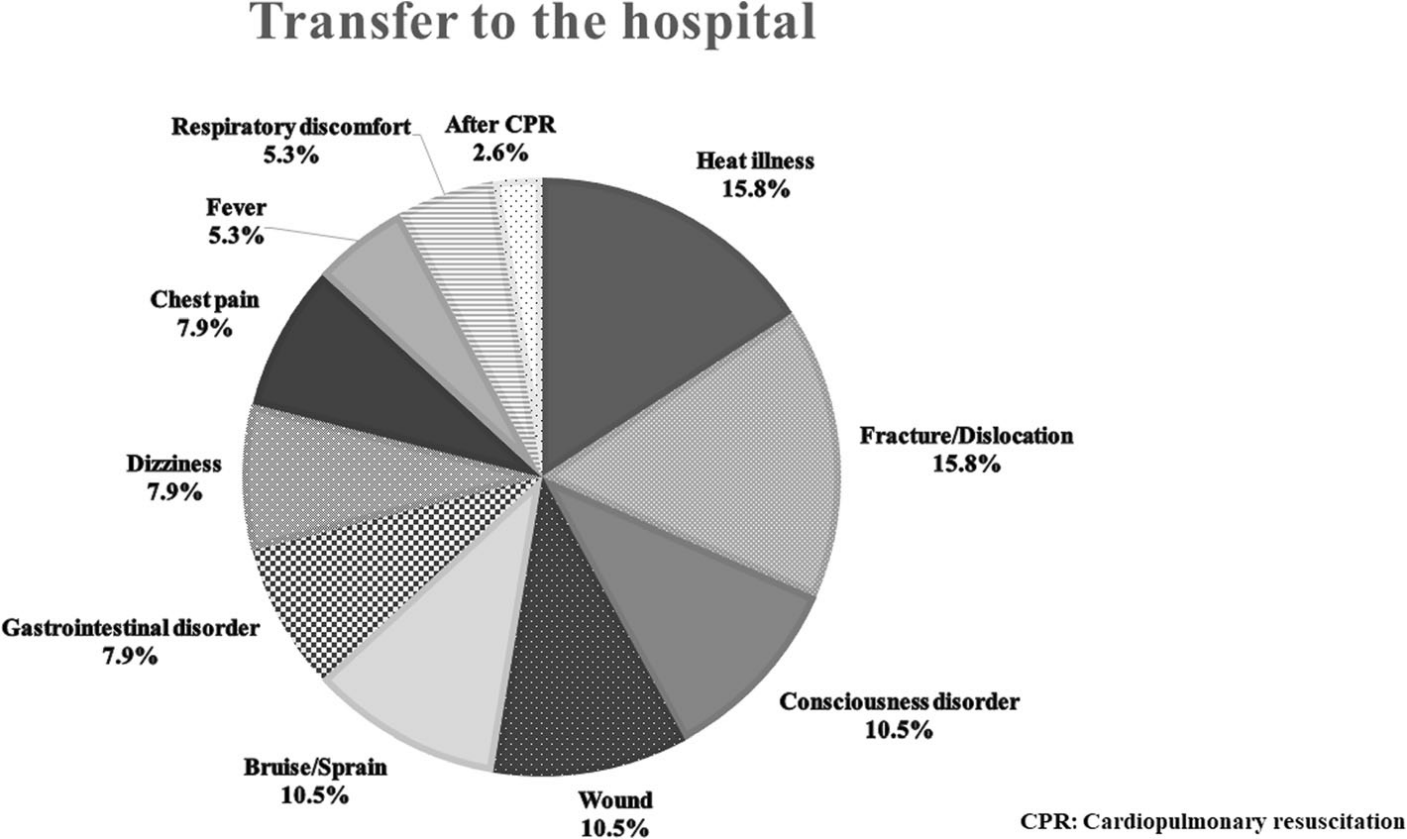
The proportion of transfers to the hospital during RWC 2019. The most common reasons for transfer to the hospital are heat illness and fracture/dislocation, with rates of 15.8% each. Consciousness disorder (including due to alcohol), wounds, and bruises/sprains show rates of 10.5% each

**Fig. 4:**
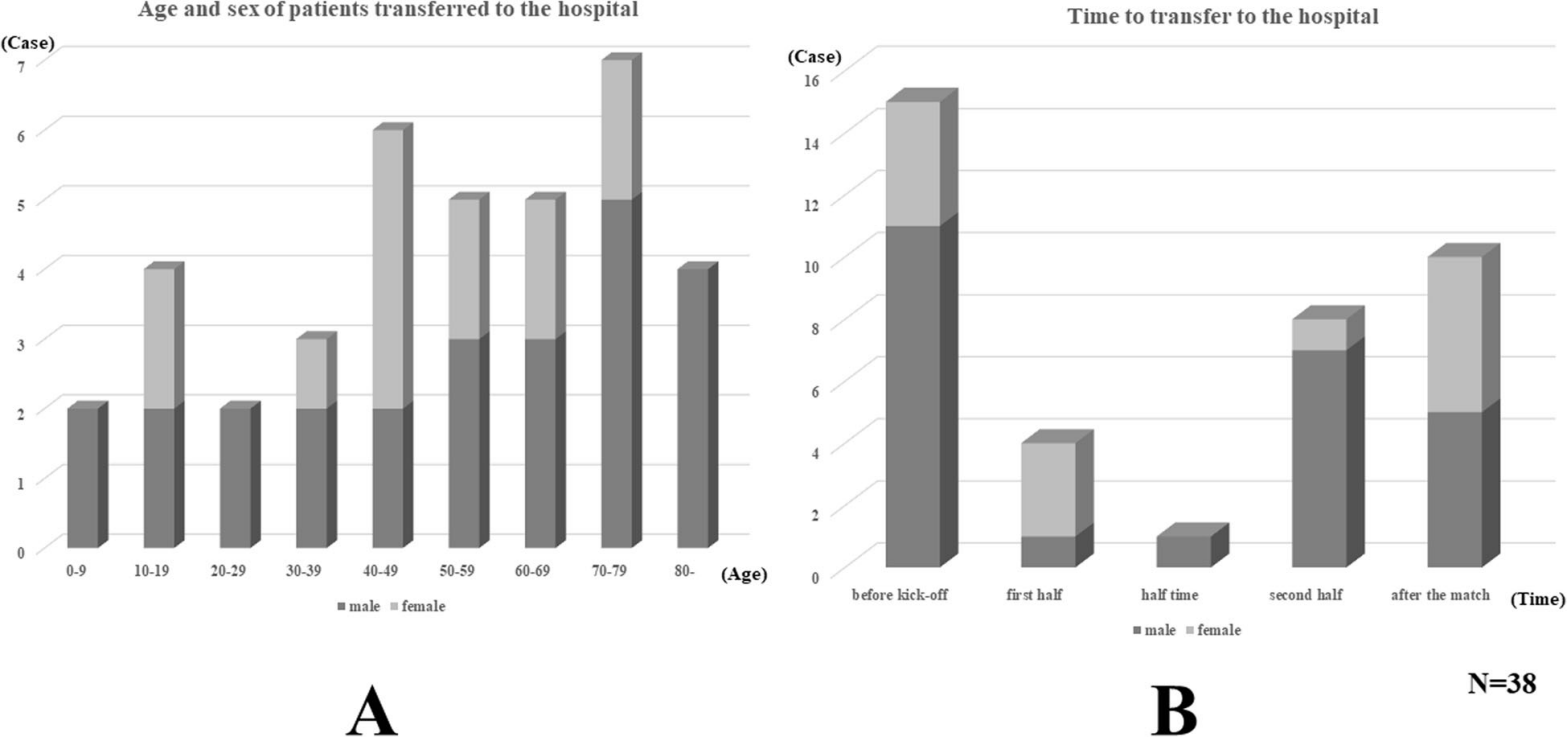
Characteristics of cases transferred to a hospital. **a** Age and sex of participants transferred to the hospital. There is no significant relationship between age and sex. **b** Time of transfer to the hospital by ambulance. There is no significant relationship between sex and time of transfer

Ten of 12 venues required ambulance transfer to the hospital at least once. The most common reasons for transfer to the hospital were heat illness and fracture/dislocation, at a rate of 15.8% each. Consciousness disorder (including due to alcohol), wounds, and bruises/sprains occurred at rates of 10.5% each (Fig. 3). Three special cases were recognized in the tournament period that were transferred from the stadium to the hospital; the first case was anaphylactic shock due to a hornet sting, the second case was a cardiopulmonary arrest with ventricular fibrillation, and the third case was dislocation of an artificial joint after total hip replacement due to a fall and rolling. For the second case, prompt and suitable CPR with an automated external defibrillator was successfully provided by the physician and nurse of the spectator medical room. The incidence rate of cardiopulmonary arrest per 10,000 attendees was 0.0059 in RWC 2019. The age and sex distribution of the 38 cases transferred to a hospital was as follows: age 0–9 years, 2 cases (male/female, 2/0); 10–19 years, 4 cases (2/2); 20–29 years, 2 cases (2/0); 30–39 years, 3 cases (2/1); 40–49 years, 6 cases (2/4); 50–59 years, 5 cases (3/2); 60–69 years, 5 cases (3/2); 70–79 years, 7 cases (5/2); and ≥ 80 years, 4 cases (4/0) (Fig. 4a). There was no significant relationship between age and sex. Time of transfer to the hospital was as follows: before kick-off, 15 cases (male/female 11/4); first half, 4 cases (1/3); half time, 1 case (1/0); second half, 8 cases (7/1); and after the match, 10 cases (5/5) (Fig. 4b). There was also no significant relationship between sex and time of transfer to the hospital.

The list of the contents of the medical kit for spectator medical room and total numbers of drugs and other medical stuffs used in the medical rooms are described in Table 7. Measuring equipment including pulse-oximeter was used appropriately in the spectator medical room. Automated external defibrillator and portable resuscitator were used once for cardiopulmonary arrest with ventricular fibrillation case.

**Table 7:** List of medical kit for spectators

1. Emergency	Usage quantity	2. Measuring equipment	Usage quantity	3. Injection/solution	Usage quantity	4. Limb injury	Usage quantity	5. Medicines	Usage quantity	6. Wound care	Usage quantity	7. Sanitary/Others	Usage quantity
Automated external defibrillator	1	Tourniquet	*	Syringe with/without needle	3	Cold spray	0	Cardiac stimulant drug	0	Swab	34	Ethyl alcohol spray	*
Portable oxygen bottle	2	Digital clinical thermometer	*	Intravenous injection needle	4	Splint for fingers/Schiene	4	Cough medicine	0	Tweezers	2	Surgical mask	0
Pulse-oximeter	*	Portable sphygmomanometer	*	Infusion solution set	4	Soft Schiene	4	Gastrointestinal drug	20	Tray	6	Towel	10
Portable resuscitator	1	Stethoscope	*	Infusion fluid	4	Triangular slings	3	Antidiarrheal drug	18	Gauze	37	Paper cup	3
		Penlight torch	*	Saline	8	Compression bandages	3	Antipyretic drug	82	Antiseptic cotton wipes	13	Soap	0
				Local anesthesia solution	0	Net bandages	4	Antipyretic drug for kids	4	Latex-coated glove	12	Tissue paper	*
				Disinfectants	*	Athletic tapes	6	Anticonvulsant drug	0	Razor	0	Wet tissue	1
						Plastic bags for ice	1	Antiallergic drug	6	Plastic bags	37	Electrolyte water bottle	44
						Easy ice bags	7	Antibiotics drug	8	Scissors	*	Napkin	37
								Antiemetic drug	21	Adhesive plaster	154		
								Antihypertensive drug	2				

*Used appropriately in each spectator medical room.

## Discussion

Mass gathering events have challenges that require special and unique medical planning. The key factor for success is the combination of preparation, planning, and communication. Previously, several trials for the preparation and planning of mass gathering sports events have been reported [11–13]. The present study focused on spectator medicine in the stadium, not for the athlete and general travellers. The most important result of this study was that most cases that visited the spectator medical room had minor to mild presentations (91.5%). These cases required minimal medical intervention, such as minor dressing, rest, icing, minimum medication, recommending hydration, and course observation. Locoh-Donou et al. also reported that the most common patient presentations at mass gathering events were mild in severity (95.97%), requiring minimal medical interventions such as dressing for abrasions or water [4]. MAGMO prepared and provided free spectator medical rooms in the stadiums. Announcement boards regarding the spectator medical rooms were set up everywhere in the stadiums. In addition, many volunteer staff, police, and security staff patrolled the stadiums and referred injured or ill persons to the nearest spectator medical room. Thus, minor or mild presentation cases were able to visit the spectator medical room easily. Moreover, even very minor cases visited the spectator medical room only to get the free medical kit, water, or medicine.

Actually, the average number of cases who visited the spectator medical room per match during RWC 2019 (9.98) was lower than the average number of medical spectator assistance cases per match in FIFA World Cup 2014 (97.2) [14]. On the other hand, for a more scientific approach to the reporting of medical issues of mass gathering events, the use of defined data points such as PPR per 10,000 and the transport to hospital rate per 10,000 has been suggested [3, 6]. In the present study, the PPR per 10,000 attendees of RWC 2019 was 2.63, while it was 0.66 in the FIFA event in South Africa [15] and 4.8 in the 5-year review of the New York State Fair [16]. The transport to hospital rate was 0.22/10,000 during RWC 2019, whereas it was 0.027/10,000 in South Africa and 2.7 per 100,000 attendees in New York. It is well-known that mass gathering medical care involves multifactorial problems. The present result showed a higher incidence rate than that of the FIFA event in South Africa. However, the South African report reviewed 6 games in a single stadium in Durban. Therefore, the rate may differ from the present rate, which included 45 matches in 12 different venues over 6 weeks. The present findings agree with the previous report of Grange et al. that an on-site physician at a large mass gathering significantly reduces the number of patients requiring transport to medical facilities [17]. It may be possible that the presence of an on-site physician, medical staff, and the development of spectator medical rooms contribute to treatment, intervention, and prompt and correct triage. Each physician in the spectator medical room was selected by the area medical officer based on clinical ability and experience. Moreover, the lead physician was selected on the match day by MAGMO. Each medical room was connected by the radio communication system, and the lead physician and MAGMO supervised medical management. Basically, decision-making in the medical room depended on each physician. Patient satisfaction could not be measured. However, when a mismatch of the physician’s specialty with the disease occurred in the medical room, the lead physician or MAGMO became involved and gave special advice for treatment or triage. This comprehensive medical management system may also contribute to suitable treatment and triage in the stadium.

It is well-known that rugby fans drink huge amounts of beer in the stadium and pubs. Moore et al. suggested that team success, but not failure, may increase aggression among supporters, and not celebration, drives post-match alcohol consumption [18]. Others have reported that alcohol was a contributing factor in up to 25% of cases of ambulance transfers [19]. However, it was difficult to identify alcohol as a factor associated with traumatic injuries.

At events held outdoors, the weather situation is one of the important factors for spectator presentations. Thus, temperature was identified as a potential contributor to spectator medicine in outdoor events. Goldberg et al. reported that, for cold weather events, transport rates increased at colder temperatures [20]. Feldman et al. reported that in Ontario’s Golden Horseshoe region during the 2015 Pan American and Parapan American Games 7827 more patients visited the physician’s office on days with a daily maximum temperature of 35 °C than of 25 °C [21]. Locoh-Donou et al. also reported in 2016 that increased PPR was strongly associated with a higher heat index (rate ratio = 1.211 per 10-unit heat index increase, *p* = 0.003) [5]. Perron et al. also reported that for every 10° increase in the heat index, three more patients per 10,000 patrons will require care, and they concluded that the heat index was strongly associated with the volume of patients at a mass gathering event [22]. Very high temperatures occur in summer in Japan [8], but the temperatures begin to fall in autumn and winter. The Japanese Ministry of Environment and the Japan Sport Association established a guideline for heat illness based on the WBGT value. Both the heat index and the WBGT value are quantitative indices of heat stress. WBGT is a measure of heat stress in direct sunlight, which takes into consideration temperature, humidity, wind speed, sun angle, and cloud cover. This differs from the heat index, which takes into consideration temperature and humidity and is calculated for shady areas [23]. In this study, WBGT was used as a measure of heat stress according to the guidance of the Japanese Ministry of Environment and the Japan Sport Association. In the guideline, the safety classification was defined as follows: safe, WBGT < 21 °C; attention, 21–25 °C; caution, 25–28 °C; alert, 28–31 °C; and dangerous, > 31 °C [24, 25]. For one of the pre RWC 2019 events, the Japan versus Fiji match, which was held in Kamaishi Stadium on July 27, the WBGT value was 31.1 °C at kick-off time. In fact, 30 patients visited the spectator medical room due to heat illness, with 2 patients transferred to the hospital by ambulance. It was a very shocking experience for RWC management and the medical officer. Therefore, the OC prepared many announcement boards in the stadium area including the parking area and provided repeat announcement messages on the screen and broadcasts to prevent heat illness at the official opening match of RWC 2019, which contained recommendations for hydration, rest, sun-shades, and so on. During RWC 2019, 7 matches had WBGT values over 25 °C in the stadium at kick-off time. For 2 of the 7 matches, the WBGT value was over 28 °C, which was divided into the alert and dangerous category. In fact, during RWC 2019, the matches that were held with a WBGT over 25 °C had significantly higher PPRs per 10,000 attendees than matches under 21 °C. On the other hand, there was no significant relationship between the WBGT value and the severity of disease or transfer to hospital in the present study. This may indicate that a higher WBGT value was associated with a higher number of patients with minor to mild presentations. Our previous experience in the pre-event and preparedness for heat illness might have helped us manage the medical care in the stadium without difficulty. The WBGT value was a useful index for medical management in a mass gathering. More careful, strict, and flexible preparation, planning, and management, especially for higher WBGT situations, are required for international mega sports events. Fortunately, there was no recognized pandemic of infections during the RWC 2019 period. Under infectious disease epidemic situation, quite another preparedness may be required for spectator medicine in the mass gathering event, such as limitation of spectator number, measuring temperature at the stadium gate, establish the special room for fever patient, and pre-check of infection. These preparedness and implementation for spectator medicine spend huge amount of labor, time, and cost. In the future, systematic and effective preparation for infection control will also be required.

### Limitations

Limitations must be taken into consideration with respect to the present study. The dataset is limited to RWC 2019. Other rugby events or other sports events were not evaluated. Although all documentation, both daily reports and individual records, were collected from the spectator medical rooms after every match, some documentation showed a lack of description of information such as the age of participants and/or the time of the visit to the medical room. Therefore, some results are shown as [unknown] category.

## Conclusion

Preparation and provision of appropriate medical services for spectators is a key factor for mass gathering events. The experience of the medical services for spectators in the stadium during RWC 2019 showed that the majority of patients that seek medical attention do so for minor complaints, which can be easily assessed and managed (91.5%), with a low transport to hospital rate (0.22 per 10,000 attendees). On the other hand, a higher WBGT situation contributes significantly to increasing the PPR (< 21 versus > 25 °C, 2.04 versus 4.27, *p* = 0.04). Careful medical preparation, management, and development of public education programs for higher WBGT situations are required. This information may help in the preparation and management of medical support for spectators in future similar international mega sports events.

## Data Availability

The datasets used and/or analyzed during the current study are available from the corresponding author on reasonable request.

## Abbreviations

RWC: Rugby World Cup
WBGT: Wet bulb globe temperature
PPR: Patient presentation rate
OC: Organizing committee
MAGMO: Medical Advisory Group Medical Officers
TMD: Tournament medical director
CPR: Cardiopulmonary resuscitation
CIs: Confidence intervals
MN: Match number
FIFA: Fédération Internationale de Football Association
HP: Home page
